# Incisional Hernia Following Ileostomy Closure: Incidence and Associated Factors in a Single-Center Retrospective Cohort Study

**DOI:** 10.7759/cureus.116994

**Published:** 2026-09-27

**Authors:** Ahmed Tawfik, Khaldoun Fozo

**Affiliations:** 1 General Surgery, Ashford and St Peter's Hospital NHS Trust, Chertsey, GBR; 2 Surgery, Surrey and Sussex Healthcare NHS Trust, Redhill, GBR

**Keywords:** body mass index, ileostomy closure, incisional hernia, obesity, stoma reversal

## Abstract

Background: Incisional hernia is a recognised complication following ileostomy closure and may lead to significant morbidity, reduced quality of life, and the need for further surgical intervention. Reported incidence varies considerably depending on the detection method and follow-up duration. This study aimed to determine the incidence of radiologically detected incisional hernia following ileostomy closure and evaluate associated patient-related factors in patients with available postoperative CT imaging.

Methodology: We conducted a single-centre retrospective cohort study at East Surrey Hospital. Adult patients undergoing ileostomy closure between January 2018 and December 2021 were identified. Patients without adequate radiological follow-up were excluded. Demographic and clinical variables, including age, sex, body mass index (BMI), smoking status, and diabetes mellitus, were collected. The primary outcome was the development of an incisional hernia at the ileostomy closure site during follow-up.

Results: One hundred patients met the inclusion criteria. Forty-five patients developed an incisional hernia, giving a radiologically detected incidence of 45%. Patients who developed a hernia had a significantly higher mean BMI than those who did not (29.2 ± 5.7 kg/m² vs. 25.5 ± 5.7 kg/m², *P* = 0.002). Obesity (BMI ≥30 kg/m²) was significantly more common among patients who developed a hernia (51.1% vs. 27.3%, *P* = 0.025). No statistically significant associations were observed for age, sex, smoking status, or diabetes mellitus.

Conclusions: Radiologically detected incisional hernia following ileostomy closure occurred in 45 (45%) patients in this cohort. Elevated BMI and obesity were significantly associated with hernia formation. These findings support preoperative risk stratification and optimisation of weight-related factors before stoma reversal.

## Introduction

Incisional hernia is a recognised complication following stoma reversal procedures and remains an important source of postoperative morbidity [1,2]. Affected patients may experience pain, cosmetic concerns, bowel obstruction, and impaired quality of life, and may require subsequent operative repair [3]. The reported incidence varies widely, ranging from approximately 7% in clinically detected series to over 30% in studies incorporating systematic radiological assessment [1,2,4,5].

Several patient-related factors have been implicated in hernia development, including obesity, diabetes mellitus, smoking, advanced age, and impaired wound healing [2,6-8]. Technical factors such as closure technique, wound infection, suture material, and prophylactic mesh reinforcement may also influence outcomes [9-13].

Despite increasing awareness of this complication, uncertainty remains regarding the relative contribution of individual risk factors, particularly in routine, non-specialist surgical practice. The aim of this study was to determine the incidence of radiologically detected incisional hernia following ileostomy closure and to evaluate associated patient-related factors in patients with available postoperative CT imaging. This study was reported in accordance with the STROCSS 2024 criteria for surgical observational studies [14].

## Materials and methods

Study design and setting

This was a single-centre retrospective cohort study conducted at East Surrey Hospital, a general district hospital in Redhill, United Kingdom. The study period spanned 1 January 2018 to 31 December 2021.

Participants

Adult patients undergoing ileostomy closure during the study period were identified from hospital operative and coding records. Patients were included if they were aged 18 years or older, underwent ileostomy closure during the study period, and had postoperative CT imaging available for review. Patients with colorectal cancer underwent CT imaging as part of routine oncological surveillance, typically at approximately six-month intervals. In patients with benign indications, CT imaging was not performed according to a predefined surveillance schedule but was undertaken according to clinical indication. All available postoperative CT scans obtained within the three-year follow-up window were assessed for the presence of an incisional hernia at the ileostomy closure site. Patients without postoperative CT imaging were excluded, most commonly those with a benign underlying indication who did not undergo routine postoperative imaging or those who were lost to follow-up.

Data collection and outcomes

Electronic medical records and radiological imaging were reviewed. Variables collected included age, sex, BMI, smoking status, and diabetes mellitus. Of the 100 patients, 85 (85.0%) had colorectal cancer and underwent routine oncological CT surveillance, typically at six-month intervals; 15 (15.0%) had benign indications and underwent CT imaging according to clinical indication rather than a predefined surveillance schedule. All available CT scans within the three-year follow-up window were reviewed, including subsequent scans after a hernia was first detected. Incisional hernia was defined as any abdominal wall fascial defect at the ileostomy closure site identified on postoperative CT imaging, based on the existing reports issued by consultant radiologists. In patients without a radiologically detected hernia, classification was based on the latest available CT examination within the follow-up period.

Statistical analysis

Continuous variables are presented as mean ± standard deviation (SD), and categorical variables as frequencies and percentages. Continuous variables were compared using the independent-samples t-test, and categorical variables using Fisher's exact test owing to the relatively small subgroup sizes. Obesity was defined as a BMI ≥30 kg/m². Statistical significance was defined as a two-sided *P*-value <0.05. Statistical analyses were performed using IBM SPSS Statistics for Windows, Version 29.0 (IBM Corp., Armonk, NY).

Owing to the modest sample size and limited number of outcome events, multivariable logistic regression was not performed. Therefore, the findings should be interpreted as associations rather than independent predictors of hernia formation.

Ethics and consent

This study was conducted as a retrospective cohort study using anonymised patient data collected during routine clinical care at East Surrey Hospital. In accordance with local institutional governance policies and United Kingdom Health Research Authority guidance, formal Research Ethics Committee approval was not required. All data were anonymised before analysis. The requirement for informed consent was waived because the study involved retrospective analysis of anonymised routinely collected clinical data and contained no patient-identifiable information.

## Results

A total of 126 patients underwent ileostomy closure during the study period. Twenty-six patients were excluded because they did not undergo postoperative surveillance CT imaging or were lost to follow-up, leaving 100 patients for the final analysis. Patient selection is summarised in Figure 1.

**Figure 1:**
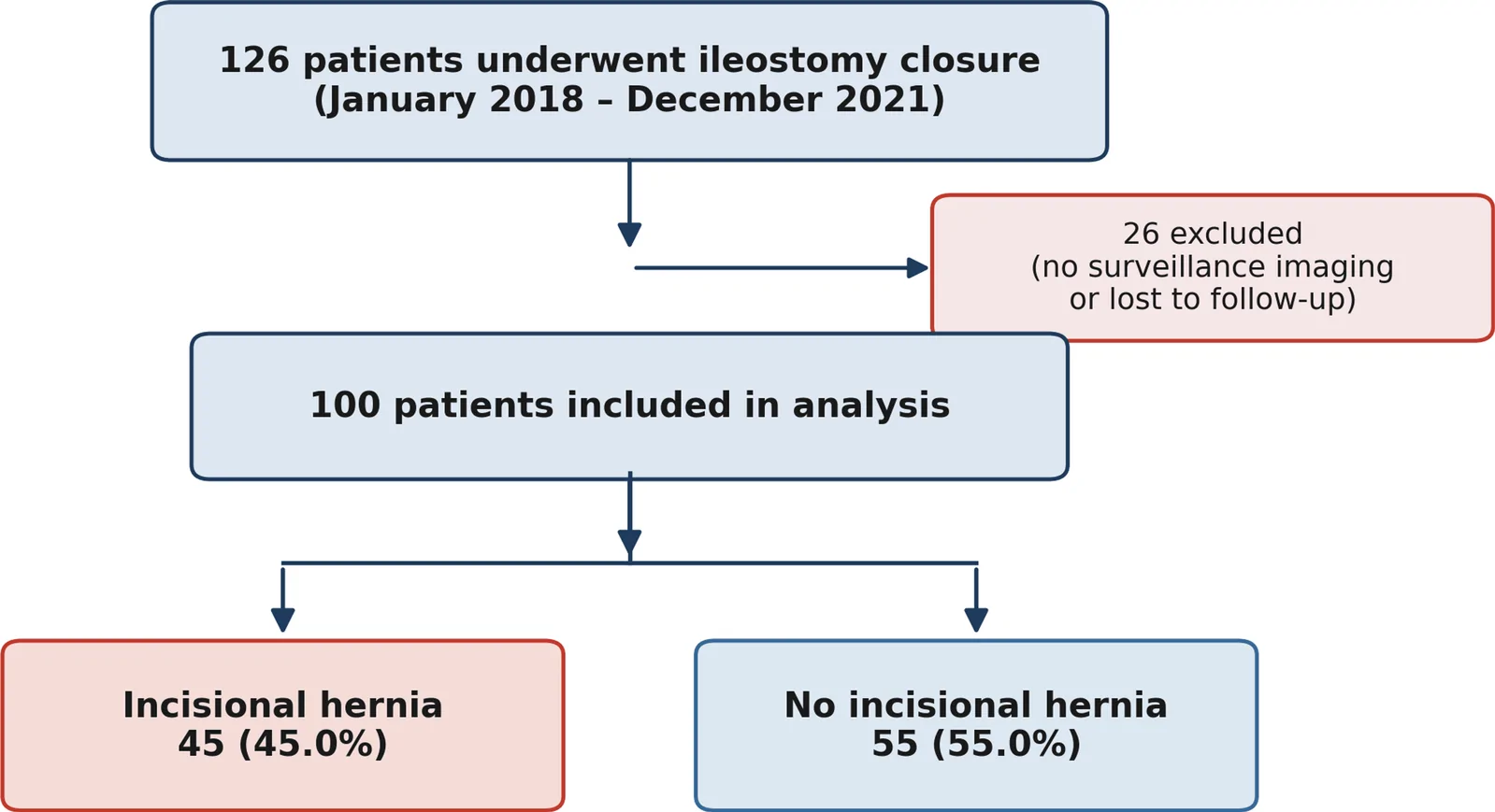
Study flow diagram showing patient identification, exclusions, and the final study cohort. The image was generated using Python 3 with the Matplotlib library (version 3.x; Matplotlib Development Team).

Baseline characteristics

The cohort had a mean age of 61.5 ± 15.0 years and comprised 55 males (55.0%) and 45 females (45.0%). The mean BMI was 27.2 ± 5.9 kg/m². Current smokers accounted for 27.0% of the cohort, and 24.0% had diabetes mellitus. Baseline characteristics are summarised in Table 1.

**Table 1:** Baseline characteristics of the cohort (n = 100). SD, standard deviation; BMI, body mass index

Characteristic	Value
Age (years) (mean ± SD)	61.5 ± 15.0
Male sex, *n* (%)	55 (55.0)
Female sex, *n* (%)	45 (45.0)
BMI (kg/m²) (mean ± SD)	27.2 ± 5.9
Current smoker, *n* (%)	27 (27.0)
Diabetes mellitus, *n* (%)	24 (24.0)

Incidence of incisional hernia

Forty-five patients (45.0%) developed an incisional hernia during follow-up, while 55 patients (55.0%) did not.

Comparison between hernia and non-hernia groups

Patients who developed an incisional hernia had a significantly higher mean BMI at the time of ileostomy closure than those who remained hernia-free (29.2 ± 5.7 kg/m² vs. 25.5 ± 5.7 kg/m², *P* = 0.002). Obesity (BMI ≥30 kg/m²) was also significantly more common among patients who developed a hernia (51.1% vs. 27.3%, *P* = 0.025). No statistically significant differences were observed between the groups with respect to age (*P* = 0.250), sex (*P* = 0.841), smoking status (*P* = 0.361), or diabetes mellitus (*P* = 1.000). These comparisons are presented in Table 2.

**Table 2:** Comparison of risk factors between patients who did and did not develop an incisional hernia. Continuous variables were compared using the independent-samples t-test, and categorical variables using Fisher's exact test. Continuous data are presented as mean ± SD and categorical data as number (%). Statistical significance was defined as *P* < 0.05.

Variable	Hernia (*n* = 45)	No hernia (*n* = 55)	Test statistic	*P*-value
Age (years) (mean ± SD)	63.4 ± 15.0	60.0 ± 14.9	*t* = 1.13	0.25
Male sex, *n* (%)	24 (53.3)	31 (56.4)	-	0.841
BMI (kg/m²) (mean ± SD)	29.2 ± 5.7	25.5 ± 5.7	*t* = 3.23	0.002
BMI ≥30 kg/m², *n* (%)	23 (51.1)	15 (27.3)	-	0.025
Current smoker, *n* (%)	10 (22.2)	17 (30.9)	-	0.361
Diabetes mellitus, *n* (%)	11 (24.4)	13 (23.6)	-	1.000

## Discussion

This study demonstrated a high incidence of incisional hernia following ileostomy closure, affecting 45% of patients during follow-up. Patients who developed a hernia had significantly higher BMI values and a greater prevalence of obesity than those who did not. This incidence exceeds many clinically reported rates but is consistent with studies employing systematic radiological assessment [1,2,4,5].

The incidence observed here is higher than that reported in many previously published clinical series. However, this finding is consistent with studies incorporating routine radiological assessment, which frequently identify asymptomatic hernias that would otherwise remain undetected during clinical follow-up. Previous systematic reviews have demonstrated substantial variation in reported incidence by detection method, emphasising the importance of standardised follow-up when comparing outcomes across studies [1,4].

An important distinction must be drawn between radiologically and clinically detected incisional hernia. In the present cohort, hernias were identified on postoperative CT, which detects many small or asymptomatic fascial defects that are not apparent on clinical examination. Such radiologically defined hernias are not necessarily of equivalent clinical importance: clinically evident hernias tend to be larger and symptomatic, and are more likely to cause pain, restriction, or bowel obstruction and to prompt operative repair [3,4]. The 45% incidence reported here should therefore be interpreted as the radiological burden of disease rather than the proportion of patients requiring intervention, and the true rate of clinically significant hernia is likely to be lower. This distinction is central to interpreting the wide variation in published incidence and underlines the need to report both the method of detection and the clinical significance of the hernias identified [1,4].

The principal finding was the significant association between increased BMI and hernia formation. Obesity was significantly more common in the hernia group. These findings are consistent with previous literature demonstrating that obesity contributes to increased intra-abdominal pressure, impaired wound healing, and increased tension on fascial closure [6,7].

In contrast, age, sex, smoking status, and diabetes mellitus were not significantly associated with hernia formation in this cohort. Although these variables have been identified as risk factors in previous studies, the present study may have lacked sufficient statistical power to detect smaller differences [2,6,8].

A strength of this study is the use of radiological assessment, which enabled identification of both symptomatic and asymptomatic incisional hernias and may provide a more complete estimate of radiologically detected hernia than studies relying solely on clinical assessment.

The study should also be interpreted in the context of several methodological limitations. As a single-centre study conducted in a general district hospital, the findings may not be fully generalisable to other institutions or healthcare systems. The relatively modest sample size limited statistical power and precluded robust multivariable analysis, meaning that residual confounding cannot be excluded. Consequently, the observed associations should be interpreted cautiously and cannot establish causation.

Recent evidence suggests that prophylactic mesh placement at the time of stoma closure may reduce subsequent incisional hernia without substantially increasing complications [9,10,13,15]. No prophylactic mesh was used in the present cohort, and abdominal wall closure was performed by different surgeons without a standardised technique. Standardisation of fascial closure and selective prophylactic mesh reinforcement may therefore warrant further evaluation [11,12,16,17]. Given the association between obesity and hernia formation observed in this cohort, targeted prehabilitation and weight optimisation may also represent practical preventive strategies. Future prospective multicentre studies should evaluate these interventions in larger cohorts using standardised operative techniques and radiological follow-up.

Limitations

This study has several limitations. First, its retrospective design introduces the possibility of selection and information bias. Second, it was conducted at a single general district hospital, which may limit the generalisability of the findings. Third, ascertainment of incisional hernia depended on the availability of postoperative CT imaging, which was performed predominantly as part of routine surveillance in patients with colorectal malignancy. Patients with benign indications did not undergo protocolised CT surveillance, and the timing and duration of their radiological follow-up therefore varied according to clinical indication. Consequently, a negative CT at the last available examination cannot exclude the subsequent development of an incisional hernia, and some late hernias may have been missed in this subgroup. Patients with benign indications who did not undergo postoperative CT imaging were also excluded, resulting in their under-representation within the cohort. These factors introduce potential selection and ascertainment bias.

The sample size was relatively modest and may have been insufficient to detect smaller differences in several risk factors, including smoking status and diabetes mellitus. Furthermore, the number of outcome events did not permit robust multivariable modelling; therefore, independent predictors of hernia formation could not be established, and residual confounding cannot be excluded.

Only a limited number of patient-related, operative, and perioperative variables were available for analysis. Abdominal wall closure was performed by different surgeons without a standardised technique. Potentially relevant factors, including anastomotic leak, cancer status, indication for stoma formation, interval to closure, suture material, surgical site infection, and other postoperative complications, were not analysed and may have influenced hernia development.

## Conclusions

Incisional hernia following ileostomy closure occurred in 45 (45%) patients in this cohort. Elevated BMI and obesity were significantly associated with hernia development, whereas age, sex, smoking status, and diabetes mellitus were not. These findings support the importance of weight optimisation and risk stratification before stoma reversal. Further prospective multicentre studies are required to validate these observations and guide preventive strategies.
